# *FADS1* rs174550 genotype and high linoleic acid diet modify plasma PUFA phospholipids in a dietary intervention study

**DOI:** 10.1007/s00394-021-02722-w

**Published:** 2021-10-31

**Authors:** Topi Meuronen, Maria A. Lankinen, Olli Kärkkäinen, Markku Laakso, Jussi Pihlajamäki, Kati Hanhineva, Ursula Schwab

**Affiliations:** 1 Institute of Public Health and Clinical Nutrition, University of Eastern Finland, PO box 1627, 70211 Kuopio, Finland; 2 School of Pharmacy, Faculty of Health Sciences, University of Eastern Finland, Kuopio, Finland; 3 Institute of Clinical Medicine, Internal Medicine, University of Eastern Finland and Kuopio University Hospital, Kuopio, Finland; 4 Department of Medicine, Endocrinology and Clinical Nutrition, Kuopio University Hospital, Kuopio, Finland; 5 Department of Life Technologies, Food Chemistry and Food Development Unit, University of Turku, Turku, Finland; 6 Department of Biology and Biological Engineering, Division of Food and Nutrition Science, Chalmers University of Technology, Gothenburg, Sweden

**Keywords:** *FADS1*, Linoleic acid, Metabolomics, Personalized nutrition, Diet, Human

## Abstract

**Introduction:**

Fatty acid desaturase 1 (*FADS1*) gene encodes for delta-5 desaturase enzyme which is needed in conversion of linoleic acid (LA) to arachidonic acid (AA). Recent studies have shown that response to dietary PUFAs differs between the genotypes in circulating fatty acids. However, interactions between the *FADS1* genotype and dietary LA on overall metabolism have not been studied.

**Objectives:**

We aimed to examine the interactions of *FADS1* rs174550 genotypes (TT and CC) and high-LA diet to identify plasma metabolites that respond differentially to dietary LA according to the *FADS1* genotype.

**Methods:**

A total of 59 men (TT *n* = 26, CC *n* = 33) consumed a sunflower oil supplemented diet for 4 weeks. Daily dose of 30, 40, or 50 ml was calculated based on body mass index. It resulted in 17–28 g of LA on top of the usual daily intake. Fasting plasma samples at the beginning and at the end of the intervention were analyzed with LC–MS/MS non-targeted metabolomics method.

**Results:**

At the baseline, the carriers of *FADS1* rs174550-TT genotype had higher abundance of long-chain PUFA phospholipids compared to the *FADS1* rs174550-CC one. In response to the high-LA diet, LA phospholipids and long-chain acylcarnitines increased and lysophospholipids decreased in fasting plasma similarly in both genotypes. LysoPE (20:4), LysoPC (20:4), and PC (16:0_20:4) decreased and cortisol increased in the carriers of rs174550-CC genotype; however, these genotype–diet interactions were not significant after correction for multiple testing.

**Conclusion:**

Our findings show that both *FADS1* rs174550 genotype and high-LA diet modify plasma phospholipid composition.

**Trial registration:**

The study was registered to ClinicalTrials: NCT02543216, September 7, 2015 (retrospectively registered).

**Supplementary Information:**

The online version contains supplementary material available at 10.1007/s00394-021-02722-w.

## Introduction

Composition of human metabolome is dependent on the genes encoding enzymes for metabolic pathways, and availability of specific substrates. Some of metabolites, precursors, or intermediates of the metabolism are derived directly from diet. These metabolites are metabolized to produce wide range of molecules which act as cellular signaling factors and structural components. Thus, the human metabolome is dependent on both diet and variants in the genes encoding enzymes for metabolism. In the current study, we aimed to investigate how genetic variation in a polyunsaturated fatty acid (PUFA) metabolism-related fatty acid desaturase 1 gene (*FADS1*) affects subjects’ metabolic profiles and responses to a high linoleic acid (LA, 18:2n6) diet.

Decreased incidence of type 2 diabetes and cardiovascular diseases are associated with higher levels of n-6 LA and long-chain n-3 PUFAs in circulation but not with long-chain n-6 arachidonic acid (AA, 20:4n6) [1–3]. However, levels of PUFAs in circulation are not solely determined by their dietary intakes. Dietary fatty acids and variation in the genes involved in fatty acid metabolism explain some of the variation in fatty acid composition of plasma lipids [4]. Dietary sources for essential fatty acids, LA and alpha-linolenic acid (ALA, 18:3n3), include plants and plant-derived oils [5]. *FADS1* encodes for fatty acid delta-5 desaturase (D5D) enzyme. Enzymatic route involving D5D is responsible for the conversion of LA and ALA to AA and eicosapentaenoic acid (EPA, 20:5n3), respectively. First, fatty acid delta-6 desaturase (D6D) converts LA and ALA, both from dietary sources, to gamma-linolenic acid (GLA, 18:3n6) and stearidonic acid (SA, 18:4n3), respectively. Fatty acid elongase 5 enzyme elongates GLA and SA to dihomo-gamma-linolenic acid (DGLA, 20:3n6) and eicosatetraenoic acid (ETA, 20:4n3), which are further desaturated by D5D to AA and EPA [6]. Estimated enzyme activity of D5D is modified by genetic variants in *FADS1*, and the conversion rate of LA to AA differs between the carriers of different genotypes [7, 8].

Allelic frequencies of *FADS* variants differ across populations [9]. *FADS* haplotype, which is associated with lower plasma proportions of AA and DHA, occurs at a frequency of 1% in African population, 97% in native Americans, and 25–50% in Europeans and Asians. This *FADS1* haplotype associates with 43% and 24% lower plasma levels of AA and DHA, respectively, compared to the homozygous haplotype with more efficient conversion [10]. In addition to human genetic ability to metabolize dietary PUFAs, the intake of PUFAs varies from country to country. The consumption of n-6 LA has been increasing during the last 100 years in the US, and currently, n-6/n-3 ratio ranges from ~ 10:1 in the US (1999) [11] to ~ 3:1 in Finland (The National FINDIET 2017 Survey). The gene expression of *FADS1* is affected by several intronic variants in the *FADS* gene cluster [12–15]. Carriers of alleles associated with higher *FADS1* expression have higher estimated activity of D5D [7, 8, 16] and thus higher rates of conversion of LA to AA, which causes differences in the fatty acid composition of plasma lipids. Cross-sectional studies indicate that genetic variants of *FADS1* have an impact on several cardiometabolic risk factors. Lower plasma concentrations of triglycerides and higher high-density lipoprotein (HDL) particles [15], especially large and very large HDL particles [17], and glucose [18], are related to the variant with higher D5D activity. These subjects are at higher risk for developing type 2 diabetes [18]. Considering the differences in the dietary intake of PUFAs across the populations and the impact of *FADS1* variants on fatty acid metabolism and metabolic traits, the interactions of this gene and diet should be studied to further reveal its effects on human health.

We have recently shown that the impact of dietary LA on high-sensitivity C-reactive protein (hs-CRP) concentration depends on the rs174550 variant of *FADS1* [16]. Mechanisms how dietary LA and genetic variants of *FADS1* are associated with the inflammatory responses are poorly understood. We applied non-targeted LC–MS metabolomics in a study, in which subjects with different *FADS1* rs174550 genotypes received a diet rich in LA for 4 weeks. We aimed to find metabolites that could explain differences seen on hs-CRP. Furthermore, we investigated the interactions between *FADS1* rs174550 genotypes and a diet rich in LA. The difference in metabolic profiles at the baseline and the rate of conversion of LA to AA potentially modulates the response to dietary PUFAs. Utilization of non-targeted metabolomics approach for fasting plasma samples allowed us to estimate the differences between endogenous metabolism of fatty acids and overall metabolome between *FADS1* rs174550-TT and -CC genotypes and the effects of the high-LA diet.

## Materials, subjects, and methods

### FADSDIET1 intervention

The full description of the FADSDIET1 intervention has been previously reported [16]. In brief, carriers of the *FADS1* rs174550 TT and CC genotypes were invited from the Metabolic Syndrome In Men (METSIM) cohort. Altogether, 59 healthy non-diabetic men with *FADS1* rs174550 TT genotype (*n* = 26) and CC genotype (*n* = 33) completed the intervention (Online Resource 1). The carriers of the CC genotype were significantly older compared to the carriers of the TT genotype, but there were no other significant differences in the clinical markers (Table 1). Carriers of the *FADS1* rs174550 TT genotype had significantly higher estimated D5D activity in plasma cholesteryl esters before the intervention [16]. Participants consumed their habitual diets throughout the intervention with the 30, 40, or 50 ml/day sunflower oil supplementation. Sunflower oil dosing was dependent on BMI. The dosing of sunflower oil supplementation was estimated as a percentage of total energy intake to ensure similar relative supplementation to all subjects despite of their BMI. The supplementation provided approximately 17–28 g LA on top of normal daily intake. Fasting plasma samples were collected before and after the 4-week intervention. A 4-day food record was collected before the intervention and a 7-day food record during the intervention. Portion sizes were weighted or estimated using household measures and pictures of portion sizes. Food records were checked by a clinical nutritionist at return. Food records were analyzed by the AivoDiet nutrient calculation software (version 2.0.2.1; Aivo Finland). The study plan was approved by the Ethical Committee of the Hospital District of Northern Savo and all the treatments were carried out according to the Declaration of Helsinki. There were no available data on the differences in the response to dietary LA between the carriers of different *FADS1* genotypes at the time of designing the study, so the power calculation was based on the effect of *FADS1* rs174550 genotype on the plasma fatty acids. Based on the cross-sectional data in the METSIM study, there is 95% power to observe significant differences (30%) between the carriers of the TT and CC genotype, *α* = 0.05, in the proportion of plasma AA with ten participants/group [16]. It is likely that a larger, > 10, sample size is needed to observe significant differences on the plasma metabolites with non-targeted metabolomics. Based on these assumptions, the sample size was estimated to 30/group.

**Table 1:** Clinical characteristics of participants at baseline according to *FADS1* rs174550 genotype

	TT (*n* = 26)	CC (*n* = 33)	*p*^1^
	Median (IQR)	Median (IQR)	
Age, years	55.0 (53.0; 56.8)	59.0 (57.0; 61.0)	8.30E-05
Body weight, kg	81.4 (73.5; 88.3)	79.7 (73.7; 83)	0.536
BMI, kg/m^2^	25.7 (23.6; 27.6)	24.9 (22.5; 26.6)	0.151
Waist circumference, cm	95.0 (89.3; 100.0)	95.4 (90.0; 98.5)	0.748
Serum fasting total cholesterol, mmol/L	5.3 (4.8; 5.6)	5.5 (4.5; 6.3)	0.292
Serum fasting HDL cholesterol, mmol/L	1.4 (1.2; 1.6)	1.4 (1.1; 1.5)	0.988
Serum fasting LDL cholesterol, mmol/L	3.4 (2.8; 3.6)	3.3 (2.7; 4)	0.861
Serum fasting triglycerides, mmol/L	1.0 (0.8; 1.3)	1.2 (1.0; 1.6)	0.055
Plasma fasting glucose, mmol/L	5.6 (5.3; 5.9)	5.7 (5.5; 6)	0.232
Plasma fasting insulin, mU/L	6.7 (5.1; 10.0)	7.9 (5.4; 9.7)	0.703
Systolic blood pressure, mmHg	129.0 (118.8; 133.8)	125.0 (118.0; 134.0)	0.652
Diastolic blood pressure, mmHg	82.5 (77.5; 86.8)	81.0 (77.0; 89.0)	0.994
Use of statins, *n* (%)	2 (8)	2 (6)	

Data are presented as median and interquartile range (IQR)

*p*^1^; Mann–Whitney’s *U* test for baseline differences between the *FADS1* rs174550 TT and CC genotypes

### Metabolomics analysis

Non-targeted liquid chromatography–mass spectrometry-based metabolic profiling analysis was performed at Afekta Technologies Ltd. (www.afekta.com) using reversed-phase (RP) and hydrophilic interaction chromatographies (HILIC) in positive and negative electrospray ionization (ESI). An aliquot of the plasma sample, 100 µL, was mixed with 400 µL of acetonitrile and mixed by pipetting. Samples were centrifuged at 18,000× *g* for 10 min at 4 °C to filter through 0.2 µm polytetrafluoroethylene filters in a 96-well plate. Small aliquots (2–5 μL) were taken from the plasma samples, mixed together in one tube, and used as the quality control sample (QC) in the analysis. The samples were analyzed by liquid chromatography–mass spectrometry (LC–MS), consisting of a 1290 Infinity Binary UPLC coupled with a 6540 UHD Accurate-Mass Q-TOF (Agilent Technologies). Zorbax Eclipse XDB-C18 column (2.1 × 100 mm, 1.8 μm; Agilent Technologies) was used for the RP separation and an Acquity UPLC BEH amide column (Waters) for the HILIC separation. After each chromatographic run, the ionization was carried out using jet stream ESI in the positive and negative mode. The collision energies for the MS/MS analysis were selected as 10, 20, and 40 V, for compatibility with spectral databases.

Data were collected by “Find by Molecular Feature” algorithm in MassHunter Qualitative Analysis B.07.00 software (Agilent Technologies, USA). The allowed ion species in ESI( +): [M + H] +, [M + Na] + , [M + K] + , [M + NH4] + , and [2M + H] + , and in ESI( −): [M − H] − , [M + Cl] − , [M + HCOO] − , [M + CH3COO] − , and [2M − H] − . Signals with height ≥ 3000 counts and ≥ 2 ions were included in the compound list. Peak spacing tolerance for isotope grouping was 0.0025 *m/z* plus 7 ppm, with isotope model for common organic molecules. We used Mass Profiler Professional (Agilent Technologies) for peak alignment. The data were combined in one.cef file after the first initial alignment, against which the original raw data were reanalyzed with compound mass tolerance was ± 15 ppm, retention time ± 0.150 min, and symmetric expansion value for chromatograms ± 35.0 ppm. Peak alignment and data cleanup were performed with Mass Profiler Professional software.

#### Data preprocessing

The signal intensities of the molecular features were corrected for the drift pattern caused by the long LC–MS run. Regularized cubic spline regression was fit separately for each signal on the QC samples and the feature abundances of all samples were normalized using the acquired drift function [19]. Prior to data filtering, the data matrix was divided into four different matrixes, genotypes, and visits separately. These matrixes were filtered with 50% rule; if metabolite is present in over 50% of any taken group, it will be selected for further analyses. Filtering reduced the percentage of missing values from 38 to 20%. Number of features decreased from 12,619 to 10,427. After filtering, the data were log10 transformed and missing values were imputed by random forest with ‘’missForest’’ R package [20]. Maximum number of iterations were set to 10. Features with mass over 1000 Da and average intensity less than 50 000 were removed. Number of features decreased from 10,427 to 3452 after filtering steps. These 3452 features were used in statistical analyses. Before multivariate statistical analyses, the data were normalized to quantiles and autoscaled.

#### Statistical analyses

Metabolite profiles at the baseline were compared with the unpaired Mann–Whitney’s *U* test and paired version was applied to compare differences between baseline and week 4 timepoints. To analyze differences on the responses to the high-LA diet between genotypes, later referred as genotype × diet interactions, metabolite measurements were rank normalized and analyzed using linear mixed effect model using R statistical software and nlme package (version 3.1). Metabolite measurement was used as a dependent variable, genotype x visit interaction, genotype, and visit were set as fixed effect, and subject identifier as a random effect. For metabolomic data, Benjamini–Hochberg FDR correction for multiple comparisons was used for *p* values and applied for all 3452 features. FDR *p* < 0.05 was considered as statistically significant and unadjusted *p* values < 0.05 as nominally significant. The nutrient intake data were analyzed with the same univariate methods than metabolomic data and *p* < 0.05 was considered as statistically significant. Partial least-squares discriminant analyses (PLS-DA) were performed with the R package “mixOmics” [21]. Pre-intervention differences in metabolic profiles between the carriers of the *FADS1* rs174550-TT and -CC genotypes were analyzed with PLS-DA. Changes within both genotypes were also analyzed with the multilevel PLS-DA, which is a paired extension of traditional PLS-DA and can be considered as a multivariate version of a traditional univariate *t* test. The performance of PLS-DA models was assessed with receiver-operating characteristics curves and area under the curve (AUC). A number of components in final models were evaluated with the function “perf”; 2, 3 and 2 components for baseline, the TT and CC genotype models. Classification error rates indicate robust models. AUC and *p* values for PLS-DA models: baseline (comp1 0.9615 1.485e-09, comp2 1 5.761e-11), TT genotype (comp1 0.8595 8.7e-06, comp2 0.9808 2.716e-09 and comp3 0.9956 8.738e-10), and CC genotype (comp1 0.8861 6.941e-08, comp2 0.9578 1.627e-10). Sizes of changes within groups during the intervention were estimated by Cohen’s *d* with the corrections for small sample sizes [22]. R packages ComplexHeatmap v. 2.2.0 [23] and ggpubr v. 0.4.0 were used for data visualization.

#### Identification of metabolites

Features with average variable importance in projection value (VIP) > 1.5 on the model for baseline differences and both multilevel models for genotypes were selected for identification. Features with unadjusted *p* value < 0.05 were selected for identification. Metabolites of interest were identified with three different levels. Level 1 identifications were matched against their mass, retention time, and MS/MS spectra ions from in-house library (commercial standards). Level 2 identification was matched against databases (MS-DIAL library version 3.96 [24], HMDB [25], Metlin [26]) and in-house library including putatively identified compounds by their mass and MS/MS fragmented ions. Level III were predicted based on the fragmentation spectra, including in silico fragmentation.

Non-targeted metabolite profiling analysis revealed metabolites affected by the diet and the genotype. When focusing on the differential features between the genotypes, there were at the baseline 369 features with VIP > 1.5 and 54 features with FDR *p* value < 0.05. For the changes observed during the intervention within the TT genotype group, the corresponding numbers were 314 and 37 and for the CC genotype 468 and 303. Total of 149 features showed nominally significant genotype × diet interaction, referring to differential response to high-LA diet between the carriers of the *FADS1* rs174550 genotypes, out of which none were statistically significant after FDR correction. Total of 58 metabolites were identified: 19, 25, and 14 for level 1, 2, and 3 identifications, respectively. List of all identified metabolites with analytical data are listed in Online Resources 2–4.

## Results

### Dietary intakes before and during the FADSDIET intervention

There were no differences in dietary intakes between the carriers of the TT and CC genotypes at the baseline or during the intervention (Table 2). Total energy (kcal), total fat (E%), MUFA (E%) and PUFA (E%), and LA (g) and vitamin E (mg) intakes increased during the intervention in response to the high-LA diet. However, positive energy balance led to no significant weight gain [16]. Absolute dietary intakes of other PUFAs (ALA, EPA, and DHA) remained unchanged.

**Table 2:** Dietary intake of nutrients before (4-day food records) and during (7-day food records) the intervention in the carriers of the *FADS1* rs174550 TT and CC genotypes

	Baseline		Intervention		*p*^1^	*p*^2^ TT	*p*^2^ CC	*p*^3^
	TT (*n* = 26)	CC (*n* = 33)	TT (*n* = 26)	CC (*n* = 33)				
	Median (IQR)	Median (IQR)	Median (IQR)	Median (IQR)				
Energy, kcal	2226 (2013; 2596)	2286 (2011; 2532)	2519 (2220; 2816)	2639 (2319; 2840)	0.601	0.003	1.79E-05	0.976
Carbohydrates, g	220.4 (183.9; 252.7)	228.1 (211.3; 279.1)	228.8 (201.0; 269.0)	246.9 (208.9; 278.5)	0.417	0.920	1.000	0.905
Carbohydrates, E%	42.1 (37.6; 46.1)	39.6 (38.3; 46)	37.0 (31.6; 41.8)	37.2 (33.6; 41.0)	0.826	0.001	4.61E-05	0.710
Protein, g	95.9 (88.4; 108.5)	102.7 (87.0; 120.2)	92.0 (79.6; 106.3)	99.2 (82.1; 108.1)	0.644	0.036	0.235	0.345
Protein, E%	17.4 (16.1; 20.4)	17.7 (15.9; 18.8)	14.5 (13.1; 16.2)	14.5 (13.6; 16.5)	0.710	8.94E-08	5.53E-07	0.175
Fat, g	86.3 (71.4; 98.8)	87.9 (71.4; 104.1)	121.7 (102.7; 135.8)	121.3 (106.9; 133.2)	0.826	7.45E-07	3.26E-09	0.720
Fat, E%	34.7 (30.1; 38.8)	35.0 (29.9; 37.8)	44 (40.4; 45.7)	42.2 (39.8; 45.0)	0.958	1.28E-06	1.25E-07	0.907
SFA, g	29.6 (24.8; 34.9)	29.1 (25.2; 37.7)	34.2 (26.9; 40.1)	33.9 (28.6; 40.7)	0.922	0.031	0.140	0.655
SFA, E%	11.9 (9.5; 14.5)	12.0 (10.4; 13.3)	12.3 (11; 13.8)	12.1 (10.0; 13.1)	0.910	1.000	0.458	0.259
MUFA, g	28.2 (23.4; 34.7)	31.5 (25.1; 38.8)	37.9 (30.9; 41.6)	37.3 (33.9; 42.5)	0.500	1.66E-04	3.40E-05	0.728
MUFA, E%	11.6 (11; 12.7)	12.5 (10.6; 13.8)	13.4 (12.5; 14.3)	13.6 (11.8; 14.2)	0.601	0.003	0.006	0.620
PUFA, g	13.8 (11.6; 19.6)	16.1 (11.6; 19.8)	36.4 (32.1; 42.3)	37.0 (34.5; 40.7)	0.631	1.49E-07	2.33E-10	0.804
PUFA, E%	5.9 (5.1; 6.6)	5.8 (5.0; 7.1)	13.2 (12.5; 14.7)	12.7 (11.6; 14.3)	0.946	5.96E-08	2.33E-10	0.818
LA, g	8.7 (7.4; 12.3)	8.8 (6.9; 11.8)	30.9 (26.9; 36.9)	30.1 (28.1; 34.6)	0.898	5.96E-08	2.33E-10	0.605
LA, E%	3.6 (3; 4.4)	3.4 (2.9; 4.4)	11.1 (10.7; 12.7)	10.5 (9.4; 12.2)	0.874	5.96E-08	2.33E-10	0.446
ALA, g	2.3 (1.8; 3.4)	2.2 (1.5; 3.1)	2.7 (1.9; 3.0)	2.3 (2.0; 3.1)	0.699	0.901	0.537	0.872
EPA, mg	75.6 (22.7; 153.2)	114.6 (24.3; 258.6)	58.2 (36.4; 167.3)	139.3 (81.4; 269.7)	0.417	0.548	0.491	0.387
DHA, mg	216.6 (69.1; 412.4)	326.1 (83.8; 735.8)	193.4 (90.6; 427.4)	358.2 (219.3; 670.9)	0.305	0.353	0.548	0.575
n-6 / n-3	3.0 (2.5; 3.5)	2.8 (2.2; 3.2)	8.6 (8; 10.1)	7.3 (6.2; 9.6)	0.183	2.98E-08	2.33E-10	0.955
LA / ALA	3.9 (3.6; 4.6)	4.1 (3.5; 4.6)	12.6 (11; 16)	13 (10.7; 14.6)	0.779	2.98E-08	2.33E-10	0.727
Vitamin E, mg	10.6 (9.7; 15.1)	11.7 (8.8; 14.7)	32.8 (28.9; 39.9)	33.8 (29.8; 37.2)	0.722	5.96E-08	2.33E-10	0.512

Data are presented as median and interquartile range (IQR)

*p*^1^; Mann–Whitney’s *U* test for baseline differences between the TT and CC genotypes, *p*^2^; paired Wilcoxon test for the changes within genotypes, *p*^3^; linear mixed effect model for significance of the responses between TT and CC genotype to high-LA diet

*SFA* saturated fatty acid, *MUFA* monounsaturated fatty acid, *PUFA* polyunsaturated fatty acid, *LA* linoleic acid, *ALA* alpha-linolenic acid, *EPA* eicosapentaenoic acid, *DHA* docosahexaenoic acid

### Differences in the metabolic profiles at baseline between the carriers of *FADS1* rs174550 genotypes

**Fig. 1:**
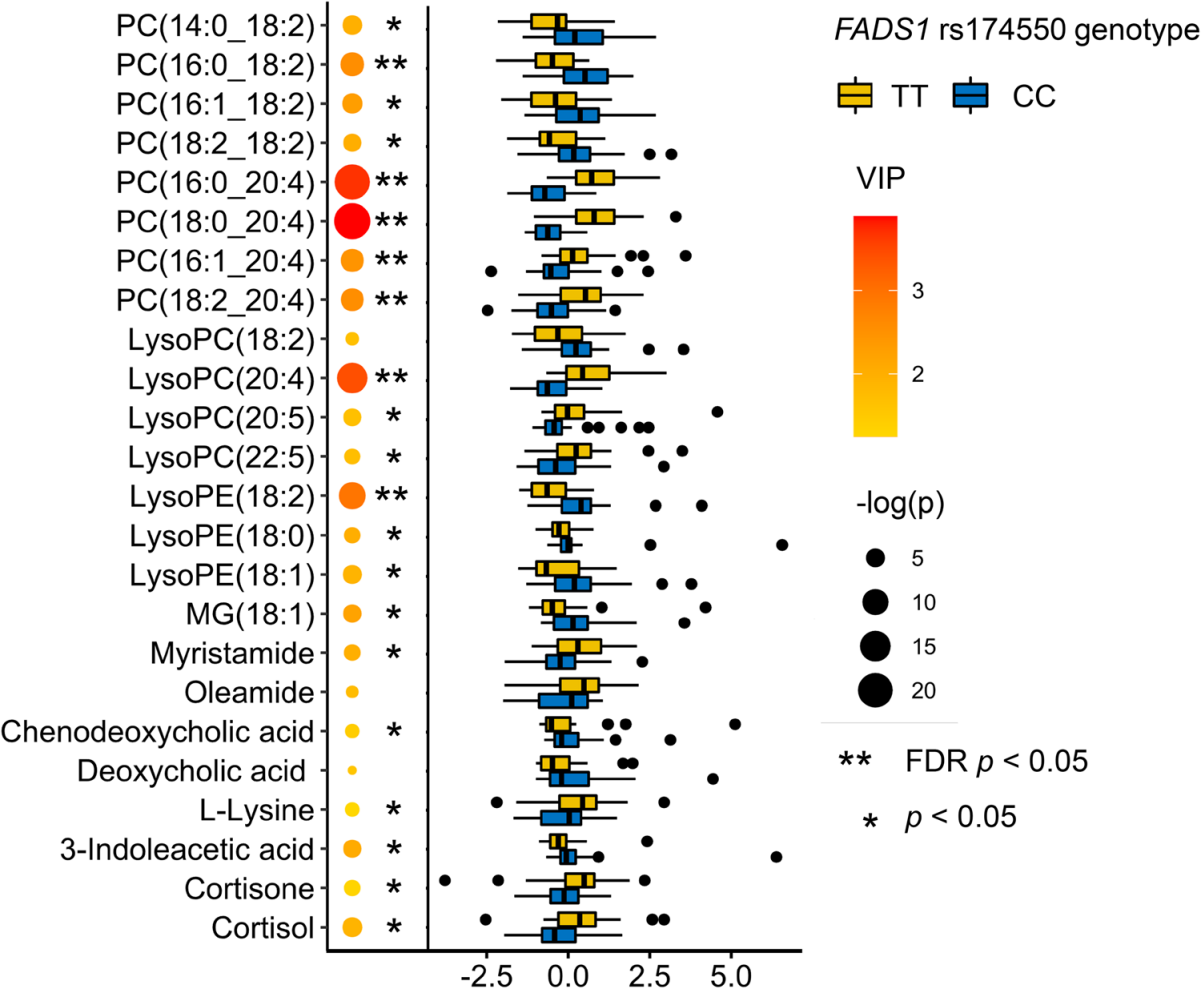
Scaled abundances of identified metabolites (*p* < 0.05 or VIP > 1.5) at the baseline according to *FADS1* rs174550 genotypes (TT = yellow, CC = blue). Size of the circle indicates − log10 transformed *p* value of the Mann–Whitney U test; color of the circles indicates the value of VIP from the PLS-DA model. Black asterisks (**) indicate significant (FDR *p* < 0.05) and (*) nominally significant (*p* < 0.05) difference at the baseline metabolite abundance between *FADS1* genotypes

The carriers of the *FADS1* rs174550-TT genotype had higher abundances of long-chain PUFA phospholipids at the baseline. Abundances of phosphatidylcholines (PCs) PC(16:0_20:4), PC(18:0_20:4), PC(16:1_20:4), PC(18:2_20:4), and lysophosphatidylcholine (LysoPC) LysoPC(20:4) were significantly (FDR *p* < 0.05) higher in the carriers of the *FADS1* rs174550-TT genotype (Fig. 1). The carriers of the rs174550-CC genotype had significantly higher abundances of PC(16:0_18:2) and lysophosphatidylethanolamine (LysoPE) LysoPE(18:2). In addition to univariate statistics, differences between genotypes on plasma metabolite composition were analyzed with multivariate PLS-DA model. The carriers of the TT genotype, due to higher D5D activity, had higher (VIP > 1.5 and *p*_unadjusted_ < 0.05) abundances of phospholipids with LC-PUFA including LysoPC (20:5) and LysoPC (22:5). The carriers of the CC genotype, with lower D5D activity, had also higher abundances of phospholipids with LA including PC(14:0_18:2), PC(16:1_18:2), and PC(18:2_18:2) (VIP > 1.5 and *p*_unadjusted_ < 0.05). Apart from fatty acids, the carriers of the TT genotype had higher abundances of amides (oleamide, myristamide) (VIP > 1.5) and steroid hormones cortisol and cortisone (VIP > 1.5 and *p*_unadjusted_ < 0.05). Identified metabolites which differed between the metabolic profiles of *FADS1* rs174550 TT and CC genotypes at baseline are shown in Fig. 1 and listed in Online Resources 3 and 4.

### Metabolic responses in plasma lipids induced by the high-LA diet

**Fig. 2:**
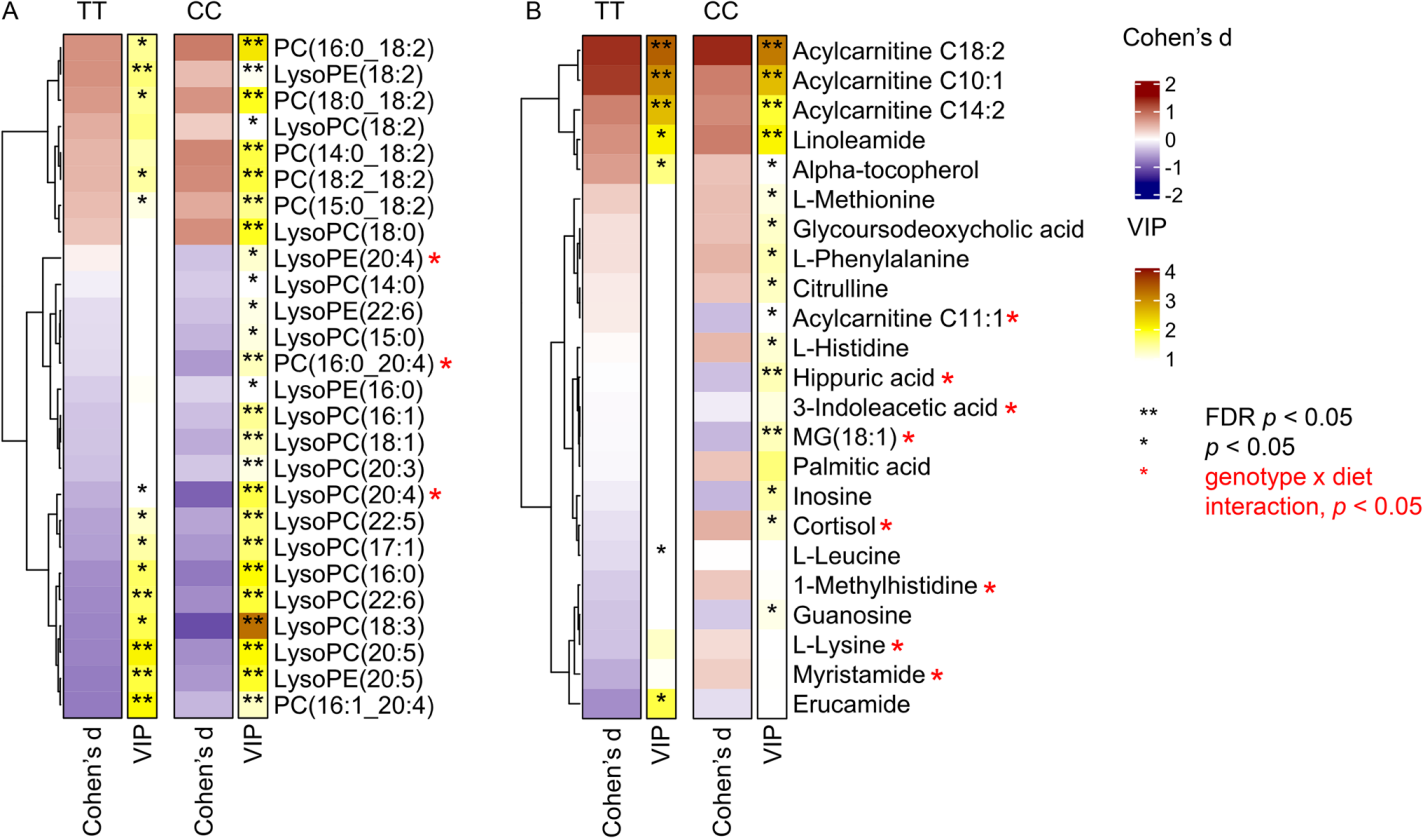
Heatmaps showing changes induced by the dietary intervention in metabolic profiles of the *FADS1* rs174550 CC and TT genotypes on **A** phospholipids and **B** other identified metabolites. Color of the cell indicates changes between baseline and week 4; red color for positive Cohen’s d value (increased metabolite) and blue for negative Cohen’s *d* (decreased metabolite). VIP values of multilevel PLS-DA models are shown with color scales and *p* value of Wilcoxon test with asterisks, ** = FDR * p* < 0.05 and * = *p* < 0.05. Red asterisk after metabolite name refers to genotype x diet interaction term *p* value < 0.05; after FDR correction, none of these were statistically significant

The 4-week high-LA diet induced changes in fatty acid metabolites in both genotypes (Fig. 2A). Abundance of LysoPE(18:2) increased significantly in both genotypes in response to the high-LA diet. Fasting plasma abundances of long-chain acylcarnitines (LCACs) C18:2, C14:2, and C10:1 increased significantly in both genotypes (Fig. 2B). There was a tendency toward increased plasma abundance of other LA phospholipids and linoleamide and alpha-tocopherol in both genotypes. Plasma abundances of PC(16:1_20:4), LysoPC(20:5), LysoPC(22:6), and LysoPE(20:5) decreased significantly in both genotypes. LysoPC(18:0) increased, but significantly only in the carriers of the CC genotype.

After FDR correction, there were no statistically significant genotype x diet interactions. In spite of similar responses seen in LA phospholipids in both genotypes, there were genotype x diet interactions (*p*_unadjusted_ < 0.05) in AA phospholipids (Fig. 2A). This finding suggests that response to high-LA diet differs between the carriers of the *FADS1* rs174550 TT and CC genotypes. Decrease in the plasma abundances of PC(16:0/20:4), LysoPC(20:4), and LysoPE(20:4) were strong for the CC genotype, whereas PC(16:0/20:4) and LysoPE(20:4) remained unchanged and LysoPC(20:4) decreased only slightly for the TT genotype. From these, PC(16:0/20:4) and LysoPC(20:4) were higher in the TT genotype carriers at baseline.

**Fig. 3:**
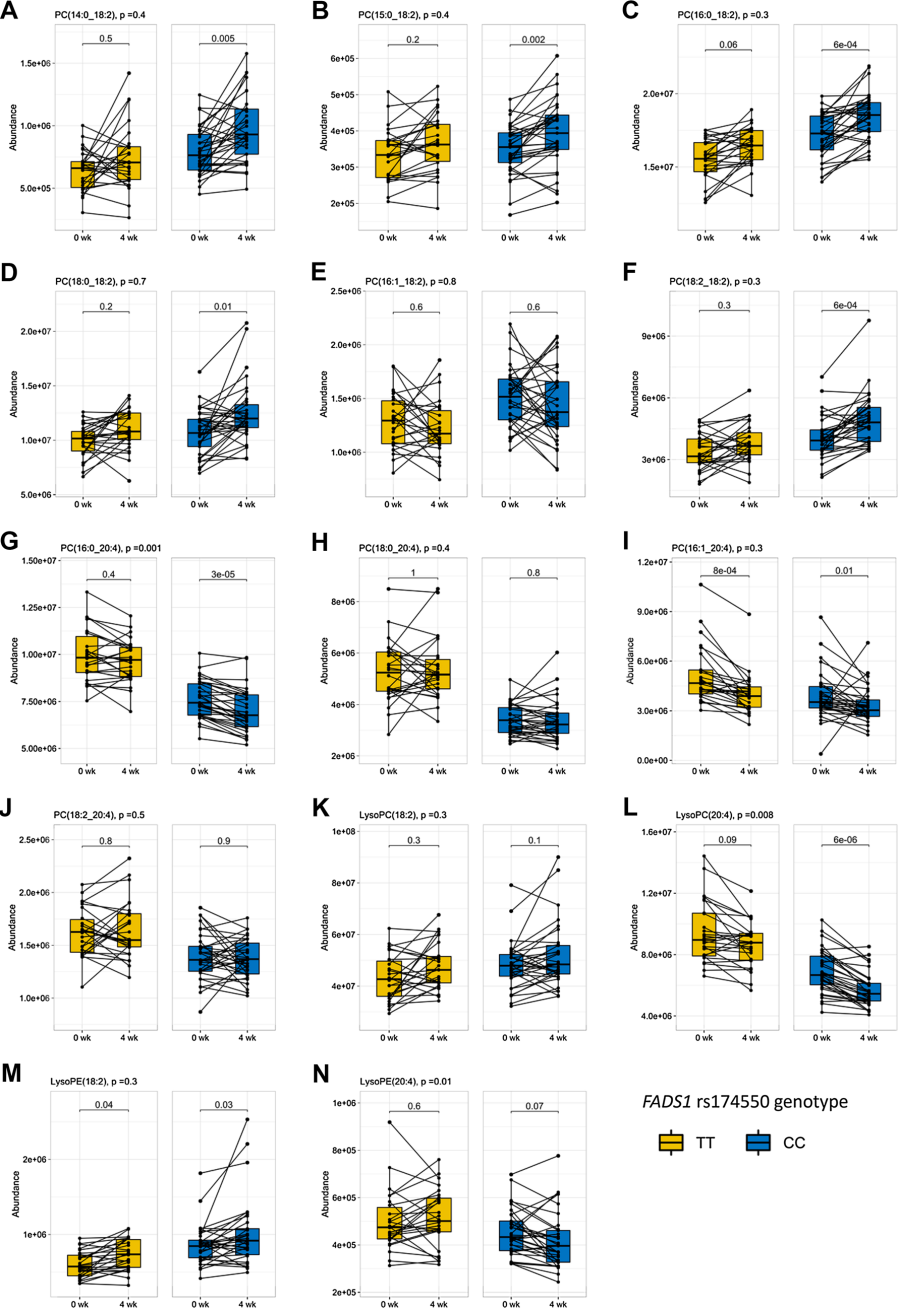
Boxplots of identified LA and AA phospholipids before and at the end of the 4-week high-LA diet according to *FADS1* rs174550 genotypes. *P* value in headline shows the genotype × diet interaction *p* value; after FDR correction, none of these were statistically significant. *P* value indicating within the genotype difference (0 week vs. 4 week) is FDR-corrected Wilcoxon test *p* value. **A** PC(14:0_18:2), **B** PC(15:0_18:2), **C** PC(16:0_18:2), **D** PC(18:0_18:2), **E** PC(16:1_18:2), **F** PC(18:2_18:2), **G** PC(16:0_20:4), **H** PC(18:0_20:4), **I** PC(16:1_20:4), **J** PC(18:2_20:4), **K** LysoPC(18:2), **L** LysoPC(20:4), **M** LysoPE (18:2), and **N** LysoPE(20:4)

We found a strong impact of *FADS1* rs174550 genotype on the plasma abundances of AA and LA phospholipids. Overall patterns of changes in LA and AA phospholipids are illustrated in Fig. 2. During the high-LA diet, AA PCs consistently decreased or remained unchanged and LA PCs increased in both genotypes (Fig. 2). The difference on plasma abundances of these phospholipids, observed at baseline, was remained even at the end of the 4-week high-LA diet. Plasma abundances of LA phospholipids at week 4 in the TT genotype carriers were mainly lower than in the CC genotype carriers at baseline (Fig. 3).

### Other metabolites associated with *FADS1* rs174550 genotype and the high-LA diet

Cortisol and cortisone were nominally lower in the carriers of *FADS1* rs174550-CC genotype compared to TT genotype carriers at baseline. There was a genotype x diet interaction (*p*_unadjusted_ = 0.004) for cortisol between the *FADS1* rs174550 genotype carriers and a nominal increase in the CC genotype carriers in response to the high-LA diet (Fig. 2B). Plasma cortisol abundance remained unchanged in the TT genotype carriers during the study. There were also other significant changes during the study which occurred only in the carriers of the CC genotype. Hippuric acid significantly decreased in the carriers of the CC genotype, but remained unchanged in the carriers of the TT genotype (genotype × diet interaction *p*_unadjusted_ = 0.048). Plasma abundances of guanosine and inosine nominally decreased and amino acids citrulline, l-Histidine, l-Methionine, and l-Phenylalanine nominally increased in the CC genotype carriers (Fig. 2B), but there were no genotype × diet interactions.

## Discussion

Our study shows that metabolite profiles in healthy middle-aged Finnish men differ between the carriers of *FADS1* rs174550-TT and -CC genotypes. Based on the 4-day food records at baseline, no differences existed in the dietary intakes of fatty acids. Despite of this, higher abundances of PC, LysoPC, and LysoPE species with an LC-PUFA, mainly AA, were found in the carriers of the rs174550-TT genotype. This finding agrees with higher activity of D5D and more efficient conversion of LA to AA in carriers of the rs174550-TT genotype. In contrast, carriers of the rs174550-CC genotype had higher abundances of LA-rich phospholipids, probably due to lower D5D enzyme activity. The differences in the absolute abundances of LA and AA phospholipids in plasma observed at baseline between the carriers of the rs174550 genotypes remained throughout the 4-week high-LA diet.

During the 4-week intervention, subjects consumed their habitual diets with sunflower oil supplementation to gain a high dose of LA. Daily supplementation increased median dietary intake of LA from ~ 9 g to ~ 30 g (~ 3.5 E% to ~ 11 E %) in both groups. The relative energy intake from PUFA increased from ~ 6 to ~ 13 E% and that of monounsaturated fatty acids (MUFA) from ~ 12 to ~ 13 E%. Although the diet was planned to be isocaloric, energy intake increased ~ 10% in both genotypes during the study. A careful documentation of the ingested sunflower oil may explain higher reported energy intake during the intervention, because no change in body weight was observed [16]. In response to the high-LA diet, in both genotype carriers, LA-rich phospholipids and LCACs (C10:1, C14:2, C18:2) increased. Fasting plasma LCAC profiles resemble the fatty acid composition of the diet [27] and in response to dietary LA LCAC(18:2) increased. The increased plasma LCAC may be a marker of disrupted lipid flux through cell and mitochondria membranes and of incomplete β-oxidation of PUFAs due to excess fatty acid input into mitochondria as it was found in women with type 2 diabetes [28]. Both LCAC C14:2 and C10:1 are chain-shortened derivates of the β-oxidation of C18:2. Higher C(10:1) may indicate reduced β-oxidation [29]. These changes in LCAC indicate changes in the energy metabolism in response to a high-LA diet in the carriers of *FADS1* rs174550-TT and -CC genotypes.

Plasma abundances of identified AA phospholipids did not significantly increase due to the 4-week high-LA diet. One possible explanation for this is that the enzymes needed for the metabolism of LA were already saturated with the substrate LA. Thus, the high-LA diet caused no significant increase in AA in plasma phospholipids in subjects consuming diets with relatively high baseline LA intakes, regardless of their *FADS1* rs174550 genotype. It has been shown that abundance of LA inhibits D6D activity in fetal human liver [30]. This finding is in agreement with a previous study (all male participants), where there were no differences in the plasma concentrations or proportions of AA between high or low LA intakes with ratios of ALA to LA 1:4 and 1:10, respectively [31]. However, a recent study [32] showed that proportions of serum AA increase modestly, regardless of the *FADS1* rs174537 genotype, in response to botanical oil rich in GLA, an n-6 PUFA, but not in response to an oil rich in LA. With a GLA enriched diet, the initial step of PUFA metabolism, conversion of LA to GLA by D6D, is bypassed.

In addition to the differences in lipid metabolites, several other classes of metabolites were affected by the genotype and dietary intervention. For example, before the intervention, carriers of the CC genotype had a nominally lower plasma abundance of cortisol and cortisone. During the intervention, cortisol nominally increased in the carriers of the CC genotype, but remained unchanged in the carriers of the TT genotype. This result supports the previous findings that the *FADS1* rs174550 genotype modifies inflammatory response, measured as hs-CRP, to dietary LA [16]. The hs-CRP concentration decreased and increased in the carriers of the TT and CC genotype, respectively. Both increasing plasma LA and n-6:n-3 ratio in response to diet are shown to increase salivary cortisol excretion in vivo [33]. The mechanism how dietary LA and the *FADS1* genotypes jointly modulate inflammatory responses remains unknown. In addition, the genotype-specific impact of the intervention on hippuric acid was an interesting observation warranting further focus on the role of genotype x diet interaction in gut microbiota function.

A recent study [12] showed that the *FADS1* rs174548, which is in strong linkage disequilibrium with rs174550 in the Finnish population [34], affects *FADS1* and *FADS2* gene expression in opposite directions in adipose tissue and skeletal muscle but not in liver. However, gene expression of *FADS1* is higher in liver compared to adipose tissue [35]. Various tissues metabolize fatty acids [36], and plasma represents the mixture of all body metabolism. These facts about whole-body fatty acid metabolism and the effects of the *FADS1* genotype on different tissues could in part explain our current results. The 4-week time might be too short for incorporation and achieving metabolic balance of fatty acids in adipose tissue and other metabolically active tissues [37]. Tissue specific effects of the *FADS1* variants together with dietary modification need to be studied to better understand mechanism behind observed changes and differences between the *FADS1* genotypes.

We observed only large differences between the carriers of the *FADS1* genotypes due to a relatively small sample size. A larger sample size would be needed to reliably observe also smaller changes in the metabolite profiles related to the *FADS1* genotype. However, we observed the genotype-specific metabolic patterns at the baseline and responses to a high-LA diet in these metabolites. Some of the changes which were seen in the CC genotype carriers (*n* = 33), but not significantly in the TT genotype carriers (*n* = 26), might arise from the lack of statistical power. The genotype-based recruitment of male participants allowed to analyze the effects of the genetic variant with high effect size on fatty acids [16]. Collected food records allowed monitoring of the diet before and during the intervention. Compliance to daily sunflower oil supplementation was good, which together with non-targeted metabolomics approach allowed comprehensive analyses of the metabolic responses to a high-LA diet on human subjects.

In conclusion, we found that the carriers of the *FADS1* rs174550-TT genotype having higher D5D activity had higher abundances of AA phospholipids. Similarly, the lower D5D activity was observed as higher abundances of LA phospholipids in the carriers of the CC genotype. The high-LA diet increased abundances of LA phospholipids. Even though an increase in LA-rich phospholipids was similar in both genotypes during the intervention, our results show that the difference in the abundances of LA- and AA-rich phospholipids between the *FADS1* rs174550 genotypes found at baseline remained at the end of the 4-week high-LA diet, and no gene–diet interaction was found.

## Supplementary Information

Below is the link to the electronic supplementary material.

Supplementary file1 (DOC 53 KB)

Supplementary file2 (XLSX 16 KB)

Supplementary file3 (XLSX 17 KB)

Supplementary file4 (XLSX 21 KB)

## Data Availability

Data described in the manuscript will not be made available, because ethical approval forbids research data sharing.

## Abbreviations

AA: Arachidonic acid
AUC: Area under the curve
D5D: Delta-5-desaturase
D6D: Delta-6-desaturase
DGLA: Dihomo-gamma-linolenic acid
ESI: Electrospray ionization
ETA: Eicosatetraenoic acid
FADS: Fatty acid desaturase
GLA: Gamma-linolenic acid
HDL: High-density lipoprotein
hs-CRP: High-sensitivity C-reactive protein
LA: Linoleic acid
LCAC: Long-chain acylcarnitine
LC–MS: Liquid chromatography–mass spectrometry
LysoPC: Lysophosphatidylcholine
LysoPE: Lysophosphatidylethanolamine
MUFA: Monounsaturated fatty acid
PC: Phosphatidylcholine
PLS-DA: Partial least-squares discriminant analysis
PUFA: Polyunsaturated fatty acid
QC: Quality control
SA: Stearidonic acid
VIP: Variable importance in projection
