# Supplementary material for: FADS1 rs174550 genotype and high linoleic acid diet modify plasma PUFA phospholipids in a dietary intervention study
Source: Eur J Nutr. 2021 Oct 31;61(2):1109–20. doi: 10.1007/s00394-021-02722-w (PMC8854246; doi:10.1007/s00394-021-02722-w)
Supplement: Supplementary file 1 — Supplementary file1 (DOC 53 KB) [file 394_2021_2722_MOESM1_ESM.doc]

**
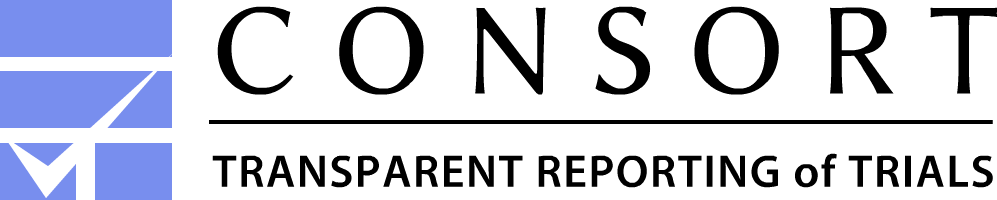
**

**CONSORT 2010 Flow Diagram**

**Allocation**

**Analysis**

**Follow-Up**

**Enrollment**

225 participants from METSIM cohort with *FADS1* rs174550 TT or CC genotypes were invited and 73 contacted the study nurse

Excluded (n= 11)

♦  Not meeting inclusion criteria (n = 4)

♦  Declined to participate (n = 5)

♦  Other reasons (n = 2)

Analysed (n = 26)
♦ Excluded from analysis (n = 0)

Lost to follow-up (n = 0)

Discontinued intervention (n = 0)

TT genotype

Allocated to intervention (n = 26)

♦ Received allocated intervention (n = 26)

Lost to follow-up (n = 0)

Discontinued intervention (n = 2)

-Busy at work (n = 1)

-Stomach problems related to sunflower oil (n = 1)

CC genotype

Allocated to intervention (n = 36)

♦ Received allocated intervention (n = 35)

♦ Did not receive allocated intervention (n = 1)

-Difficulties in blood sampling (n = 1)

Analysed (n = 33)
♦ Excluded from analysis (n = 0)

Allocated to the intervention (n = 62)

FADS1 rs174550 genotype and high linoleic acid diet modify plasma PUFA phospholipids in a dietary intervention study

European Journal of Nutrition

Topi Meuronen^1^, Maria A. Lankinen^1^, Olli Kärkkäinen^2^, Markku Laakso^3^, Jussi Pihlajamäki^1,4^, Kati Hanhineva^1,5,6^, Ursula Schwab^1,4^

^1^Institute of Public Health and Clinical Nutrition, University of Eastern Finland, Kuopio, Finland

^2^School of Pharmacy, Faculty of Health Sciences, University of Eastern Finland, Kuopio, Finland

^3^Institute of Clinical Medicine, Internal Medicine, University of Eastern Finland and Kuopio University Hospital, Kuopio, Finland

^4^Department of Medicine, Endocrinology and Clinical Nutrition, Kuopio University Hospital, Kuopio, Finland

^5^Department of Life Technologies, Food Chemistry and Food Development unit, University of Turku, Turku, Finland

^6^Department of Biology and Biological Engineering, Division of Food and Nutrition Science, Chalmers University of Technology, Gothenburg, Sweden

Corresponding author:

Topi Meuronen

Email: topi.meuronen@uef.fi
